# The discovery and characterization of K‐563, a novel inhibitor of the Keap1/Nrf2 pathway produced by *Streptomyces* sp

**DOI:** 10.1002/cam4.1949

**Published:** 2019-02-08

**Authors:** Ran Hori, Kozo Yamaguchi, Hidetaka Sato, Miwa Watanabe, Kyoko Tsutsumi, Susumu Iwamoto, Masayuki Abe, Hideyuki Onodera, Satoshi Nakamura, Ryuichiro Nakai

**Affiliations:** ^1^ R&D Division Kyowa Hakko Kirin Co., Ltd. Sunto Shizuoka Japan; ^2^ School of Life Science and Technology Tokyo Institute of Technology Yokohama Kanagawa Japan

**Keywords:** anti‐cancer agent, drug resistance, Keap1/Nrf2 pathway, NSCLC, *Streptomyces* sp

## Abstract

Keap1/Nrf2 pathway regulates the antioxidant stress response, detoxification response, and energy metabolism. Previous reports found that aberrant Keap1/Nrf2 pathway activation due to Kelch‐like ECH‐associated protein 1 (Keap1) mutations or Nuclear factor E2‐related factor 2 (Nrf2) mutations induced resistance of cancer cells to chemotherapy and accelerated cell growth via the supply of nutrients. Therefore, Keap1/Nrf2 pathway activation is associated with a poor prognosis in many cancers. These previous findings suggested that inhibition of Keap1/Nrf2 pathway could be a target for anti‐cancer therapies. To discover a small‐molecule Keap1/Nrf2 pathway inhibitor, we conducted high‐throughput screening in Keap1 mutant human lung cancer A549 cells using a transcriptional reporter assay. Through this screening, we identified the novel Keap1/Nrf2 pathway inhibitor K‐563, which was isolated from actinomycete *Streptomyces* sp. K‐563 suppressed the expression of Keap1/Nrf2 pathway downstream target genes or the downstream target protein, which induced suppression of GSH production, and activated reactive oxygen species production in A549 cells. K‐563 also inhibited the expression of downstream target genes in other Keap1‐ or Nrf2‐mutated cancer cells. Furthermore, K‐563 exerted anti‐proliferative activities in these mutated cancer cells. These in vitro analyses showed that K‐563 was able to inhibit cell growth in Keap1‐ or Nrf2‐mutated cancer cells by Keap1/Nrf2 pathway inhibition. K‐563 also exerted synergistic combinational effects with lung cancer chemotherapeutic agents. An in vivo study in mice xenotransplanted with A549 cells to further explore the therapeutic potential of K‐563 revealed that it also inhibited Keap1/Nrf2 pathway in lung cancer tumors. K‐563, a novel Keap1/Nrf2 pathway inhibitor, may be a lead compound for development as an anti‐cancer agent.

## 1 INTRODUCTION

Chemotherapy is a principal treatment for cancer patients, but some cancers can develop resistance to chemotherapy. Such resistance to chemotherapy is one of major clinical problem frequently observed during the treatment of many malignant tumors.^1^, ^2^, ^3^, ^4^ Therefore, overcoming chemotherapy resistance is clinically necessary.

Major chemotherapeutic agents, such as cisplatin and etoposide, produce reactive oxygen species (ROS).^5^, ^6^, ^7^ Nuclear factor E2‐related factor 2 (Nrf2) is a major activator for the defense protection against ROS‐induced apoptosis through the transcription of genes involved in scavenging ROS and excreting xenobiotic metabolites.^8^, ^9^, ^10^, ^11^, ^12^ Under normal conditions, Kelch‐like ECH‐associated protein 1 (Keap1) regulates the concentration of Nrf2 by proteasomal degradation.^8^, ^9^ However, the aberrant activation of the Keap1/Nrf2 pathway by gene mutation or epigenetic regulation have been reported in many cancers, including lung cancer,^13^, ^14^, ^15^, ^16^ prostate cancer,^17^ gallbladder cancer,^18^, ^19^ cervical cancer,^20^ epithelial ovarian cancer,^21^ and pancreatic cancer.^22^ Indeed, Keap1 and Nrf2 mutations have been reported in 3%‐19% and 7%‐11% of lung cancers, respectively.^23^ Moreover, many reports have found that the aberrant activation of the Keap1/Nrf2 pathway was associated with a poor prognosis in cancers with Keap1‐ or Nrf2‐ mutations, including lung cancers, gallbladder cancers, and epithelial ovarian cancers.^16^, ^18^, ^21^ Keap1 and Nrf2 mutations cause the aberrant activation of the Keap1/Nrf2 pathway through dysfunctional Keap1‐Nrf2 interaction and suppression of the Nrf2 degradation.^13^, ^16^ It has been suggested that the activation of the Keap1/Nrf2 pathway upregulates the detoxification system, which induces drug unresponsiveness or drug resistance.^13^, ^16^, ^19^, ^24^ In Keap1‐ or Nrf2‐mutated human cancer cells, genes involved with the antioxidant response have been found to be highly expressed, resulting in the acquisition of resistance to chemotherapy. From these reports, the inhibition of the Keap1/Nrf2 pathway is important for improving the drug sensitivity in cancers resistant to chemotherapy.

It was also recently reported that Nrf2 plays a role in cellular metabolism.^25^ Cancer cells reprogram the cellular metabolism to acquire more necessary nutrients in a nutrient‐deprived environment, for example, a low‐oxygen environment, and proliferate more efficiently^26^ Therefore, the inhibition of the Keap1/Nrf2 pathway was also predicted to be important for inhibiting cancer metabolism, which may lead to tumor progression inhibition.

In efforts to develop an effective therapy for such Keap1/Nrf2 pathway‐activated cancers, several small‐molecule compounds have been reported as Keap1/Nrf2 pathway inhibitors.^27^, ^28^, ^29^, ^30^, ^31^, ^32^, ^33^ These compounds have shown enhanced sensitivity to other anti‐cancer drugs and inhibited tumor growth in Keap1/Nrf2 pathway‐activated cancer models both in vitro and in vivo. Based on these reports, Keap1/Nrf2 pathway inhibitors appear to be attractive agents for treating such Keap1/Nrf2 pathway‐activated tumors.

In the present study, to identify effective Keap1/Nrf2 pathway inhibitors for cancer therapy, we performed a cell‐based screening assay using a Keap1 mutant non‐small cell lung carcinoma cell line.

## 2 MATERIALS AND METHODS

### 2.1 Structural elucidation of K‐563 purified from *Streptomyces* sp. 3728‐17

The extraction and purification of K‐563 from *Streptomyces* sp. 3728‐17 strain is described in Data S1.

The molecular formula of K‐563 was determined by high‐resolution electrospray ionization‐mass spectrometry (ESI‐MS) analysis with the following operating conditions: column, Acquity UPLC BEH C_18_ (2.1 mm i.d. × 30 mm length; Waters, Milford, MA); column temperature, 40°C; mobile phase A, 20 mmol/L ammonium acetate buffer (pH 9.0); mobile phase B, acetonitrile; elution program, 5% B (0 minutes)‐95% B (5 minutes)‐95% B (9 minutes); flow rate, 0.4 mL/min; LC‐MS system, Acquity H‐class (Waters), and Synapt G2 HDMS (Waters); ionization mode, ESI; desolvation temperature, 250°C; source temperature, 120°C; capillary voltage, 2 kV; and cone voltage, 20 V. The nuclear magnetic resonance (NMR) spectra of K‐563 were obtained using an AVANCE500 NMR instrument (Bruker, Billerica, MA) with the following operating conditions: resonant frequency, ^1^H 500.23 MHz; ^13^C 125.78 MHz; temperature, 30°C; solvent, 0.02 mol/L NaOD/CD_3_OD (1/1) mixed solution; internal standard, ^1^H (3.3 ppm) of CHD_2_OD and ^13^C (49.0 ppm) of CD_3_OD.

### 2.2 Cell lines

The human lung cancer cell line A549 and the human normal lung cell line BEAS‐2B were obtained from American Type Culture Collection (ATCC). The human lung cancer cell line LK‐2 and the human gallbladder cancer cell line TGBC24TKB were obtained from Riken BioResource Center. A549 cells were grown in Ham's F‐12K (Kaighn's) Medium with 10% fetal bovine serum (FBS). LK‐2 cells were grown in RPMI1640 with 10% FBS. TGBC24TKB cells were grown in DMEM, high glucose, pyruvate with 10% FBS. BEAS‐2B cells were grown in BEGM BulletKit. Cells were cultured at 37°C in humidified air with 5% CO_2_. All of the cell lines were authenticated by a short tandem repeat assay at the Japanese Collection of Research Bioresources.

### 2.3 Reporter cell lines

The pGL4.28 plasmid containing the ARE sequence (pGL4.28‐ARE Luc) and the pGL4.15 plasmid containing E‐cadherin promoter region (pGL4.15‐E‐cadherin promoter Luc) were constructed by Kyowa Hakko Kirin Co., Ltd. The pGL4.28‐ARE‐Luc vector or the pGL4.15‐E‐cadherin promoter‐Luc vector was transfected into A549 cells (A549/ARE‐Luc cells and A549/E‐cad promoter‐Luc cells, respectively), and hygromycin was added to the cells for stable cell line selection. A single clone with a high reporter activity was selected for further experiments.

### 2.4 siRNA transfection

Nrf2 siRNAs were purchased from Qiagen (Venlo, the Netherlands) (SI03246950) and Thermo Fisher Scientific, (Waltham, MA) (HSS181505). Negative control siRNAs were also purchased from Qiagen and Thermo Fisher Scientific. All siRNAs were transfected into A549 cells with Opti‐MEM I Reduced Serum Medium (Thermo Fisher Scientific) and Lipofectamine RNAiMAX Transfection Reagent (Thermo Fisher Scientific). SI03246950 was used to construct a reporter screening system, and HSS181505 was used for the analysis of the effects of Keap1/Nrf2 pathway inhibition.

### 2.5 Luciferase reporter assay

A549/ARE‐Luc cells or A549/E‐cad promoter‐Luc cells were treated with siRNAs or K‐563 in 96‐ or 384‐well plates. After siRNA treatment for 72 hours or K‐563 treatment for 18 hours, luminescence was measured with Steady‐Glo reagent (Promega, Madison, WI) using a Top count NXT (PerkinElmer, Waltham, MA).

### 2.6 Real‐time reverse transcription polymerase chain reaction

After siRNA treatment for 24‐48 hours or K‐563 treatment for 24 hours, total RNA was extracted from cells using an RNeasy plus kit (Qiagen), following the manufacturer's instruction. The total RNA was reverse‐transcribed using VILO reagent (Thermo Fisher Scientific). Real‐time reverse transcription polymerase chain reaction (RT‐PCR) was performed with TaqMan Gene Expression Assay (Thermo Fisher Scientific), and TaqMan Fast Universal PCR Master Mix (Thermo Fisher Scientific) or TaqMan Fast Advanced Master Mix (Thermo Fisher Scientific). The transcript levels were determined using the ABI PRISM or Applied Biosystems 7500 Fast Real‐Time PCR system (Thermo Fisher Scientific). The level of each mRNA was normalized to the level of *GAPDH* mRNA.

### 2.7 Western blotting

After K‐563 treatment for 24 hours, the cells were lysed in NP40 lysis buffer (Thermo Fisher Scientific) with protease inhibitor cocktail (Sigma‐Aldrich, St. Louis, MO). These lysate samples were then loaded onto a SuperSep Ace gel (Wako Pure Chemical Industries, Osaka, Japan) with Lane Marker Reducing Sample Buffer (Thermo Fisher Scientific) and separated by electrophoresis. The proteins were transferred to a polyvinylidene fluoride membrane using a semi‐dry transfer apparatus. The membranes were incubated with anti‐Nrf2 antibody (Abcam, Cambridge, UK), anti‐Heme Oxygenase 1 (HO‐1) antibody (Abcam) or anti‐GAPDH antibody (Sigma‐Aldrich) as a primary antibody. After being washed, the membranes were incubated with anti‐rabbit IgG, HRP‐Linked F (ab’)_2_ fragment (Donkey) (GE Healthcare, Chicago, IL) or anti‐mouse IgG, HRP‐Linked F (ab’)_2_ fragment (Sheep) (GE Healthcare) as a secondary antibody. Then, the membrane was exposed Supersignal West Femto Maximum Sensitivity Substrate (Thermo Fisher Scientific) or Supersignal West Pico Chemiluminescent Substrate (Thermo Fisher Scientific). The target proteins were detected using an Amersham Imager 600 system (GE Healthcare).

### 2.8 Glutathione level quantification

After siRNA treatment for 72 hours or K‐563 treatment for 24 hours, the Glutathione (GSH) levels in cells were measured using a GSH/GSSH‐Glo Assay Kit (Promega) or GSSH/GSH Quantification Kit (Dojindo, Kumamoto, Japan) according to the manufacturer's instructions. Either the luminescence (with the GSH/GSSH‐Glo Assay) or absorbance (with the GSSH/GSH Quantification Kit) were measured as the readout using a Top count NXT (PerkinElmer) or a SpectraMax 340PC (Molecular Devices, San Jose, CA), respectively. The GSH levels were normalized by the values observed in the negative control siRNA‐treated sample for Nrf2 siRNA or the DMSO‐treated sample for K‐563.

### 2.9 ROS detection

After siRNA treatment for 72 hours or K‐563 treatment for 24 hours, the ROS production was measured using CellROX Deep Red Reagent (Thermo Fisher Scientific) by flow cytometry according to the manufacturer's instructions. The ratio of the mean fluorescence intensity (MFI) in each sample to the MFI in the negative control siRNA‐treated sample or DMSO‐treated sample was calculated to quantify the ROS production activity.

### 2.10 Cell growth inhibition assay and the analysis of the combination effects

Cells were seeded into 96‐well plates and then treated with compounds the next day. After 72 hours of incubation, XTT reagent (Roche Diagnostics, Risch‐Rotkreuz, Switzerland) was added to the cells. After incubation at 37°C for the indicated time points, the formation of formazan dye from tetrazolium salt XTT was measured using a SpectraMax 340PC (Molecular Devices).

### 2.11 Tumor xenograft experiment

All animal studies were performed in accordance with the Standards for Proper Conduct of Animal Experiments at Kyowa Hakko Kirin Co., Ltd., under the approval of the company's Institutional Animal Care and Use Committee. Male severe combined immunodeficient (SCID) mice (C.B‐17/Icr‐scid/scidJcl, 5 weeks old) were purchased from CLEA Japan. A549 cells were suspended in PBS and inoculated subcutaneously in the shaved area of the rear right flank of SCID mice at 1 × 10^7^ cells/animal. The tumor volumes of A549 cells in xenograft mice were measured at 20 days after inoculation. The mice were divided into groups of two animals each with similarly sized A549 tumors in the flank. The average tumor size in each group ranged from 163.3 to 319.1 mm^3^ (mean ± standard error: 231.7 ± 7.3 mm^3^).

At the day of grouping, K‐563 was administered subcutaneously to the mice twice a day for 1 day. After the mice were sacrificed at 16 hours after the last administration, the tumor in the flank was collected and frozen in liquid nitrogen. Tumor total RNA was extracted using a Maxwell 16 LEV simplyRNA Tissue Kit (Promega) according to the manufacturer's instructions. The total RNA was reverse‐transcribed using VILO reagent (Thermo Fisher Scientific). RT‐PCR was performed as described before.

### 2.12 Statistical analyses

The IC_50_ values of reporter activity inhibition and GI_50_ values of cell growth inhibition were calculated using a four‐parameter logistic model of XLfit 5.3.1.3 (ID Business Solutions). The combination index (CI) was calculated by the CI‐isobologram equation,^34^ using the Calcusyn software program 2.0 (BIOSOFT) based on the data of the anti‐proliferative activity in cells treated with K‐563, a chemotherapy agent (cisplatin or etoposide) or a combination of both compounds. Through this method, CI <1 was defined as synergism.

## 3 RESULTS

### 3.1 K‐563 from *Streptomyces* sp. 3728‐17 inhibited the ARE‐luciferase reporter activity

To screen for compounds that selectively inhibit the Keap1/Nrf2 pathway, we developed a high‐throughput screening system using a reporter assay in Keap1 mutant human lung cancer A549 cells.^13^ First, A549 cells with the reporter plasmid for the Nrf2 promoter region, ARE (A549/ARE‐Luc cells) and A549 cells with the reporter plasmid for E‐cadherin promoter (A549/E‐cad promoter‐Luc cells) were constructed for the primary and counter screening, respectively.

**Figure 1:**
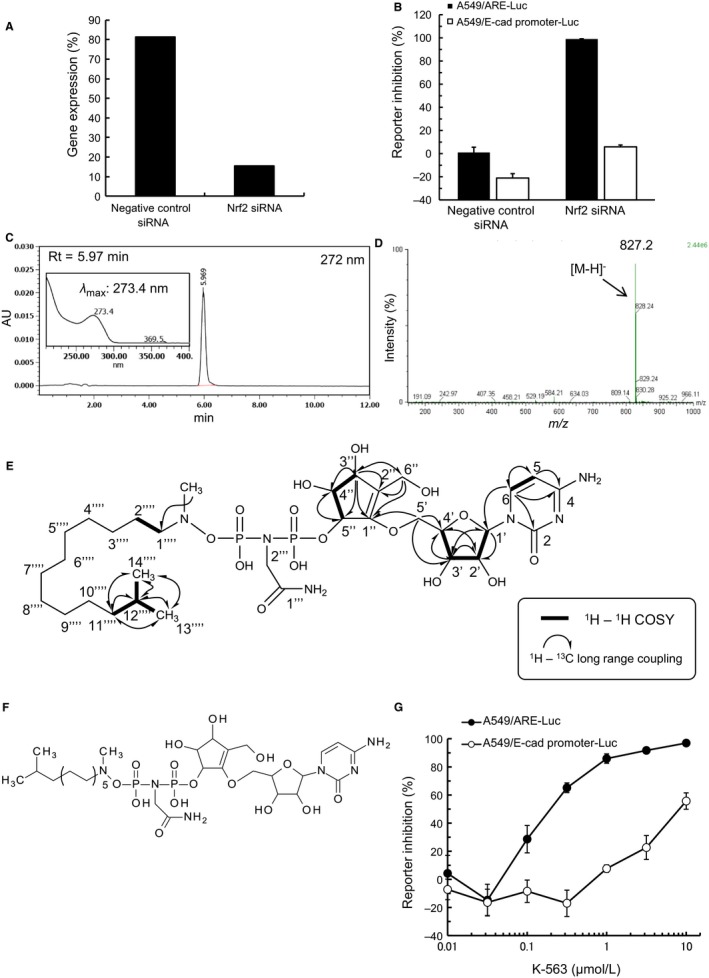
Chemical structure of K‐563 and reporter inhibition of K‐563. A, A549 cells were treated with 10 nmol/L siRNA for 24 h and collected for a real‐time RT‐PCR analysis. The mRNA expression of the *NFE2L2* gene was normalized to the expression of *GAPDH* mRNA. B, A549/ARE‐Luc cells and A549/E‐cad promoter‐Luc cells were transfected with 10 nmol/L siRNA. After 72 h of treatment, the luciferase reporter activity was measured. Each column represents the mean + SD of triplicate experiments. C and D, The purity and molecular weight of K‐563 were analyzed by HPLC and LC‐MS. The HPLC conditions are summarized in Data S1. The spectrum inserted in the HPLC chromatogram (C) shows the UV absorption of K‐563. E, The ^1^H‐^1^H COSY and HMBC correlations of K‐563. Interpretation of the ^1^H‐^1^H DQF COSY NMR data of K‐563 identified five isolated proton spin systems, and the HMBC spectrum demonstrated several long‐range H‐C couplings. F, The chemical structure of K‐563. G, A549/ARE‐Luc cells and A549/E‐cad promoter‐Luc cells were treated with K‐563. After 18 h, the luciferase reporter activity was measured. Each point represents the mean ± SD of triplicate experiments

After the construction of these reporter cells, we validated the assay system by siRNA for *NFE2L2*, encoding Nrf2 (Figure 1A), which suppressed the reporter activity of A549/ARE‐Luc cells but did not suppress the reporter activity of A549/E‐cad promoter‐Luc cells (Figure 1B). This suggested that inhibition of transcriptional activity of Nrf2 was able to suppress reporter activity only in the A549/ARE‐Luc cells in this constructed reporter assay. Thus, we performed high‐throughput screening using this reporter assay.

Through this screening with more than 10 000 culture broth samples, we found that only the culture broth of strain 3728‐17, which was isolated from a moss sample collected in Aoyama Highland, Iga, Mie, Japan, selectively inhibited the reporter activity in A549/ARE‐Luc cells. The 16S rDNA sequence of the strain 3728‐17 showed 99.7% homology to actinomycete *Streptomyces* sp. HBUM 171258 (EF608467), with no alternative candidates indicated. Consequently, the strain 3728‐17 was classified as *Streptomyces* sp.

We purified the active compound K‐563 from the culture broth by activity‐guided column chromatography as described in Doc S1. Based on LC‐MS analysis of the isolated K‐563, the UV λmax 273.4 nm (Figure 1C) and the molecular weight 828 (Figure 1D) were observed. This indicated that K‐563 was an analogue compound of 1100‐50^35^ and EM2487^36^, ^37^, ^38^ according to a database, Dictionary of Natural Products Version 22:1 (CRC press). The structure of K‐563 was finally elucidated by spectroscopic data analyses described below.

The physico‐chemical properties of K‐563 are summarized in Table 1. The molecular formula was determined to be C_32_H_58_N_6_O_15_P_2_ mainly based on the observed deprotonated molecule (found: *m/z* 827.3365 [M‐H]^−^, calculated: 827.3357) according to a negative ion mode high‐resolution ESI‐MS analysis. The ^1^H NMR (500 MHz) and ^13^C NMR (125 MHz) signal assignments are listed in Table 2. K‐563 was identified as an EM2487 analogue by comparing the mass and NMR data with those of 1100‐50 and EM2487. The cytidine moiety was confirmed by the correlations observed in ^1^H‐^1^H DQF COSY and the HMBC spectra of K‐563 (Figure 1E and Figure S1). ^1^H‐^13^C long‐range coupling between 5′‐H_2_ and C1′′ revealed the connection site of cytidine and cyclopentene. ^1^H‐^13^C long‐range coupling between the methyl proton signal at *δ* 2.74 and C1*''''* confirmed the presence of the *N*‐methyl group. Thus, the chemical structure of K‐563 was elucidated, as shown in Figure 1E,F. K‐563 inhibited the luciferase reporter activity of the Keap1/Nrf2 pathway with an IC_50_ value of 0.19 μmol/L in A549/ARE‐Luc cells, while the IC_50_ value in A549/E‐cad promoter‐Luc cells was 8.9 μmol/L, ca. 50‐fold greater than that in A549/ARE‐Luc cells (Figure 1G). Therefore, we identified this novel compound K‐563 as a selective Keap1/Nrf2 pathway reporter inhibitor.

**Table 1:** Physico‐chemical properties for K‐563

	K‐563
Formula	C_32_H_58_N_6_O_15_P_2_
High resolution ESI‐MS	[*m/z*, (M‐H)^‐^]
Found	827.3365
Calculated	827.3357
Δ(Da/ppm)	0.0008/0.97
Appearance	White powder
UV λmax(nm) (50% acetonitrile)	273.4

**Table 2:** NMR data for K‐563, 1100‐50 and EM2487

	K‐563		1100‐50		EM2487	
Position	d_C_ ppm (multiplicity, *J *= Hz)	d_H_ ppm (multiplicity, *J *= Hz)	d_C_ ppm (multiplicity, *J *= Hz)	d_H_ ppm (multiplicity, *J *= Hz)	d_C_ ppm (multiplicity, *J *= Hz)	d_H_ ppm (multiplicity, *J *= Hz)
2	158.4	‐	167.4	‐	152.5	‐
4	167.2	‐	158.6	‐	166.2	‐
5	96.9	6.04 (d, 7.6)	97.0	6.00 (d, 7.5)	103.0 (d)	5.84 (d, 7.9)
6	142.3	7.93 (d, 7.6)	142.6	7.86 (d, 7.5)	142.4 (d)	7.91 (d, 7.9)
1′	91.0	5.93 (d, 3.6)	91.2	5.88 (d, 3.6)	90.4 (d)	5.98 (d, 4.6)
2′	75.8	4.19 (m)	76.0	4.14 (dd, 3.6, 5.2)	75.7 (d)	4.24 (dd, 5.2, 4.6)
3′	69.7	4.35 (dd, 5.6, 5.8)	70.0	4.29 (br.t, 5.6)	70.3 (d)	4.42 (dd, 5.2, 5.2)
4′	83.5	4.24 (m)	83.7	4.17 (m)	84.8 (d)	4.22 (ddd, 5.2, 2.7, 2.4)
5′	69.1	4.21 (d, 11.2) 4.49 (m)	69.5	4.17 (m) 4.44 (m)	69.5 (t)	4.51 (dd, 11.6, 2.7) 4.27 (dd, 11.6, 2.4)
1′′	154.9 (d, 7.4)	‐	155.0 (d, 8.0)	‐	155.2	‐
2′′	121.2	‐	121.4	‐	121.4	‐
3′′	71.6	4.65 (dd, 1.1, 6.0)	71.8	4.61 (dd, 1.4, 6.0)	71.9 (d)	4.69 (dd, 5.8, 1.0)
4′′	76.4	4.15 (dd, 4.2, 6.0)	76.6	4.11 (dd, 4.2, 6.0)	77.0 (d)	4.38 (ddd, 5.8, 4.0, 2.5)
5′′	80.5	5.35 (m)	80.7 (d, 4.9)	5.28 (m)	81.0 (d)	5.48 (br.m)
6′′	55.0	4.11 (dd, 1.2, 12.5) 4.27 (d, 12.5)	55.2	4.06 (dd, 1.4, 12.5) 4.23 (d, 12.5)	55.4 (t)	4.31 (d, 12.2) 4.20 (dd, 12.5)
1′′′	179.1	‐	179.1	‐	178.7	‐
2′′′	51.3	3.8 ‐ 4.0 (br.m)	51.3 (br.t, 2.9)	3.80 (m), 3.87 (m)	51.7 (t)	3.9 ‐ 4.1 (br.m)
1′′′′	62.7 (d, 0.86)	2.7 (br) 3.2 (br.m)	53.7 (d, 4.7)	2.98 (br.t, 7.2)	63.2 (t)	2.98 (br.m)
2′′′′	26.6	1.48 (br.m)	27.4	1.43 (m)	27.3 (t)	1.61 (br.m)
3′′′′‐10′′′′	27.9 ‐ 30.3	1.24 (m)	28.0 ‐ 30.5	1.19 (m)	31.1 ‐ 28.6	1.28 ‐ 1.39 (br.m)
11′′′′	39.7	1.13 (m)	39.9	1.08 (m)	40.3 (t)	1.21 (br.m)
12′′′′	28.6	1.48 (m)	28.8	1.43 (m)	29.2 (d)	1.56 (t, 6.5)
13′′′′	22.9	0.84 (d, 6.7)	23.1	0.79 (d, 6.8)	23.1 (q)	0.91 (d, 6.5)
14′′′′	22.9	0.84 (d, 6.7)	23.1	0.79 (d, 6.8)	23.1 (q)	0.91 (d, 6.5)
15′′′′	‐	‐	‐	‐	‐	‐
16′′′′	‐	‐	‐	‐	‐	‐
*N*‐CH_3_	46.5	2.74	‐	‐	46.7 (q)	2.81 (br.)

Approximately 1 mg of K‐563 was dissolved in 0.02 mol/L NaOD/CD_3_OD (1/1).

^1^H (3.3 ppm) of CHD_2_OD and ^13^C (49.0 ppm) of CD_3_OD were used as the internal standard.

### 3.2 K‐563 inhibited the Keap1/Nrf2 pathway in Keap1 mutant cancer A549 cells

**Figure 2:**
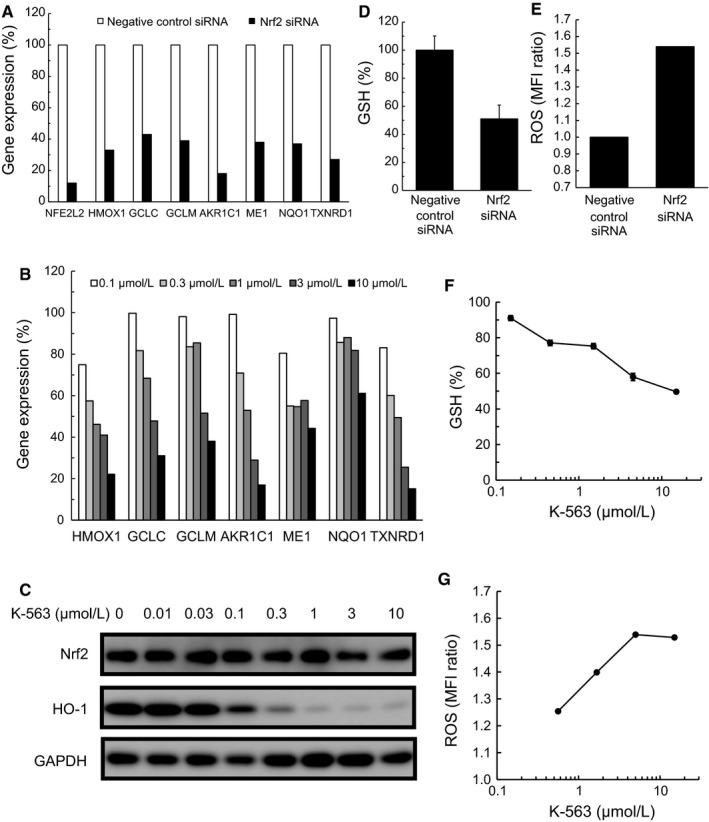
The inhibitory effects of K‐563 on Keap1/Nrf2 pathway in A549 cells. A, A549 cells were treated with 10 nmol/L siRNA for 48 h and collected for a real‐time RT‐PCR analysis of the Keap1/Nrf2 pathway downstream genes. B, A549 cells were treated with K‐563 for 24 h and collected for a real‐time RT‐PCR analysis of the Keap1/Nrf2 pathway downstream genes. Each column represents the mean of duplicate experiments. C, A549 cells were treated with K‐563 for 24 h and collected for a Western blot analysis of the Nrf2 and HO‐1 protein expression. D, A549 cells were treated with 20 nmol/L siRNA for 72 h, and the Glutathione (GSH) levels were measured. The GSH levels were normalized to those of the negative control siRNA‐treated cells. Each column represents the mean + SD of triplicate experiments. E, A549 cells were treated with 10 nmol/L siRNA for 72 h, and the reactive oxygen species (ROS) production was measured. The mean fluorescence intensity (MFI) was normalized to that of the negative control siRNA‐treated cells. F, A549 cells were treated with K‐563 for 24 h, and the GSH levels were measured. The GSH levels were normalized to those of the vehicle‐treated cells. Each point represents the mean ± SD of triplicate experiments. G, A549 cells were treated with K‐563 for 24 h, and the ROS production was measured. Each point represents the relative MFI, as described previously

To assess the Keap1/Nrf2 pathway inhibition exerted by K‐563, the inhibitory effect on the expression of the Keap1/Nrf2 pathway downstream target genes in A549 cells was firstly evaluated by real‐time RT‐PCR. Results showed that K‐563 inhibited the gene expression of heme oxygenase 1 (*HMOX1*), glutamate‐cysteine ligase synthetase catalytic subunit (*GCLC*), glutamate‐cysteine ligase synthetase modifier subunit (*GCLM*), aldo‐keto reductase family 1 member C1 (*AKR1C1*), malic enzyme 1 (*ME1*), NAD(P)H: quinone oxidoreductase 1 (*NQO1*), and thioredoxin reductase 1 (*TXNRD1*), all of which have been previously reported as Keap1/Nrf2 pathway downstream target genes^10^, ^11^, ^12^ and were inhibited by Nrf2 siRNA (Figure 2A), in a dose‐dependent manner (Figure 2B).

Next, the protein expression inhibition of the Keap1/Nrf2 pathway was evaluated by Western blotting. K‐563 inhibited the expression of HO‐1 protein, a Keap1/Nrf2 pathway downstream protein encoded by *HMOX1*, in a dose‐dependent manner (Figure 2C). In contrast, the Nrf2 protein levels at more than 0.01 μmol/L of K‐563‐treatment were almost equal to those at vehicle treatment. From these results, it was suggested that K‐563 had no effect on Nrf2 expression for Keap1/Nrf2 pathway inhibition. Furthermore, no inhibitory effects on Nrf2 nuclear translocation or Nrf2‐ARE binding were observed (Figure S2). These analysis findings suggest that K‐563 exerted an inhibitory effect on the transcriptional activity of Nrf2 via a different mechanism from that which suppressed the Nrf2 expression, Nrf2 translocation, and binding to ARE.

The Keap1/Nrf2 pathway regulated the detoxification and the antioxidative stress response by controlling the intracellular GSH and ROS levels. Nrf2 siRNA suppressed the GSH levels and increased the ROS production in A549 cells (Figure 2D,E). Therefore, to further clarify the Keap1/Nrf2 pathway inhibition exerted by K‐563, the effects of K‐563 on the GSH and ROS production were evaluated. K‐563 suppressed the GSH levels in a dose‐dependent manner in A549 cells (Figure 2F) and inhibited GSH levels by 50% at the maximum dose of 15 μmol/L. It was suggested that K‐563 inhibited GSH levels through the suppression of GSH synthesis pathway‐related genes, such as *GCLC* and *GCLM* (Figure 2B). The ROS production was increased in a dose‐dependent manner in K‐563‐treated A549 cells (Figure 2G), and K‐563 increased the ROS production by 1.5 times at the maximum dose of 15 μmol/L.

These findings along with the effects of K‐563 on Keap1/Nrf2 pathway suggest that the increase in the ROS production was promoted by the suppression of antioxidative stress response‐related proteins expression, such as HO‐1 (Figure 2C), and low GSH levels (Figure 2D) induced through Keap1/Nrf2 pathway inhibition by K‐563. Based on these results, K‐563 was defined as a novel inhibitor of the Keap1/Nrf2 pathway.

### 3.3 K‐563 inhibited the expression of Keap1/Nrf2 downstream genes and cell growth in Keap1‐ and Nrf2‐mutated cancer cell lines

We confirmed that K‐563 inhibited the Keap1/Nrf2 pathway in Keap1‐mutated human lung cancer A549 cells. We therefore next investigated whether or not K‐563 inhibited the Keap1/Nrf2 downstream gene expression and exerted cell growth inhibition in other Keap1‐ or Nrf2‐mutated human cancer cell lines.

**Figure 3:**
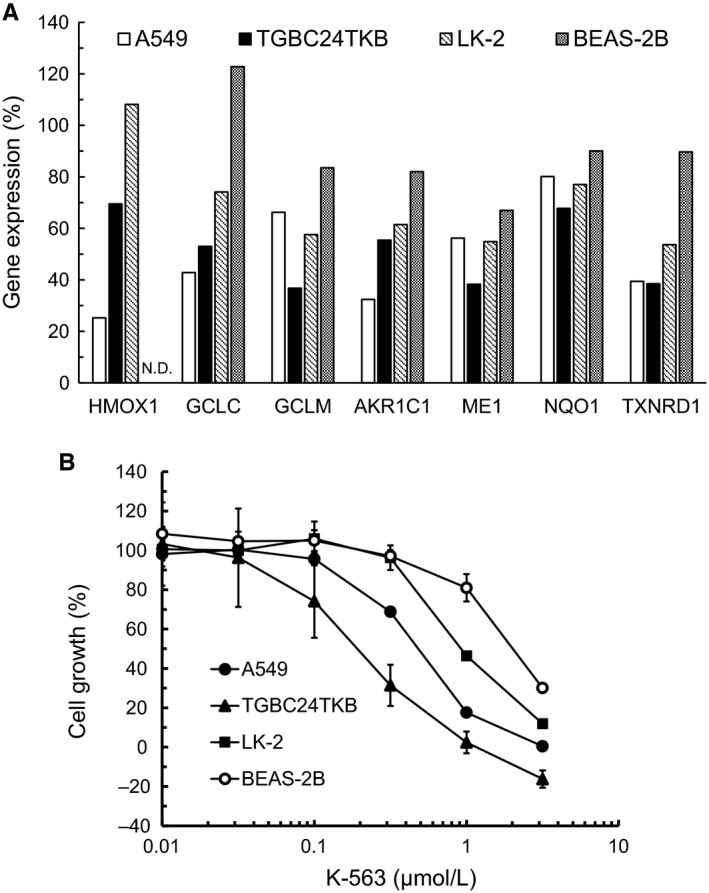
Keap1/Nrf2 pathway inhibition and cell growth inhibition of the Keap1‐ or Nrf2‐mutated cancer cell lines and human normal lung cells treated with K‐563. A, Cells were treated with 15 μmol/L of K‐563 for 24 h and collected for a real‐time RT‐PCR analysis. Each column represents the mean of duplicate experiments. N.D.: not detected. B, Cells were treated with K‐563 for 72 h, and an XTT assay was performed. Each point represents the mean ± SD of triplicate experiments

LK‐2 is a Nrf2‐mutated human lung cancer cell line,^16^ and TGBC24TKB is a Keap1‐deficient human gallbladder cancer cell line.^19^ The expression of Keap1/Nrf2 pathway downstream target genes in Keap1‐ or Nrf2‐mutated cancer cells treated with K‐563 were measured by real‐time RT‐PCR. K‐563 inhibited the expressions of Keap1/Nrf2 pathway downstream target genes (*HMOX1*,* GCLC*,* GCLM*,* AKR1C1*,* ME1, NQO1,* and *TXNRD1*) in not only A549 cells but also TGBC24TKB and LK‐2 cells (Figure 3A). In addition, the suppression of these genes expression in A549, TGBC24TKB, and LK‐2 cells was stronger than that in BEAS‐2B cells, which are human normal lung cells with low Nrf2 activity (average expression of the seven genes: 49%, 51%, 70%, and 89%, respectively).^13^ Therefore, these results indicate that K‐563 inhibited the expression of Keap1/Nrf2 pathway downstream target genes in human cancer cells harboring Keap1 or Nrf2 mutations.

Next, the inhibitory effects of K‐563 on the cell growth of Keap1‐ or Nrf2‐mutated cells were examined. K‐563 showed anti‐proliferative activity in A549 cells with a GI_50_ value of 0.48 μmol/L (Figure 3B). K‐563 inhibited the cell growth of A549 cells at a concentration that was consistent with the downregulation of the Keap1/Nrf2 pathway downstream target gene expression (Figures 2B and 3B). Taken together, these findings suggest that K‐563 inhibited cell growth via Keap1/Nrf2 pathway inhibition, such as GSH production inhibition and ROS production (Figure 2F,G). K‐563 also showed anti‐proliferative activity in TGBC24TKB and LK‐2 and cells with GI_50_ values of 0.18 and 0.95 μmol/L, respectively (Figure 3B). In contrast, the GI_50_ value of BEAS‐2B was 2.2 μmol/L. Given these results, K‐563 inhibited not only the Keap1/Nrf2 pathway, but also the cell growth more strongly in A549, TGBC24TKB, and LK‐2 cells than in BEAS‐2B cells (Figure 3A,B). These results thus suggest that Keap1/Nrf2 pathway inhibition mediated by K‐563 contributed to cell growth inhibition in human cancer cells harboring Keap1 or Nrf2 mutations, and that this inhibitory effect might be based on the selectivity of K‐563 for the Keap1/Nrf2 pathway.

### 3.4 K‐563 exerted combinatory anti‐proliferative effects when administered with chemotherapeutic agents

**Figure 4:**
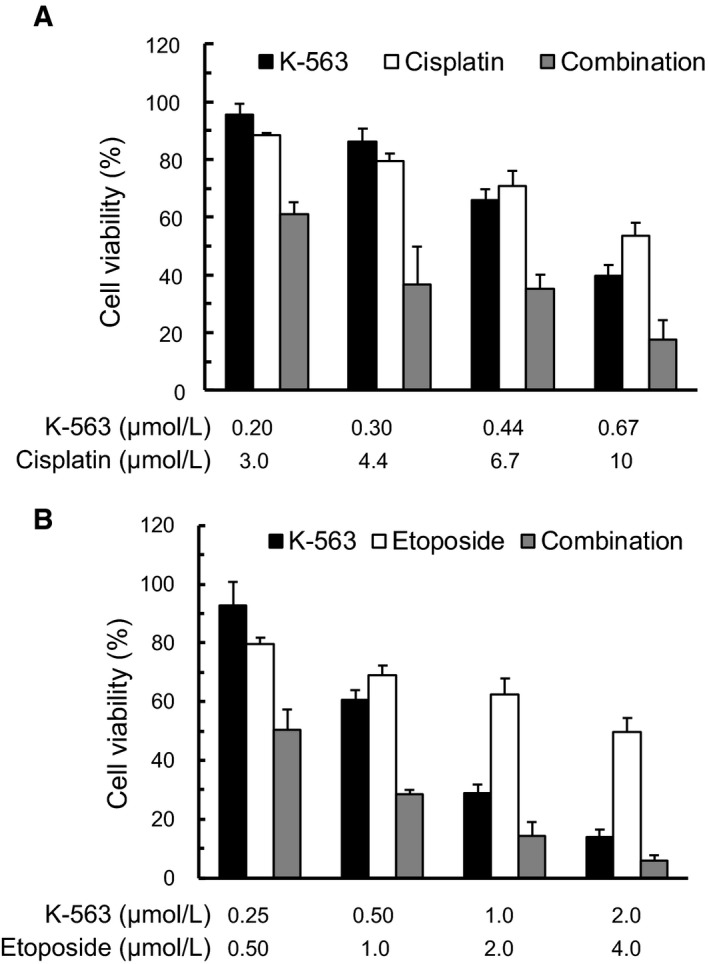
The combination effect of K‐563 plus cisplatin or etoposide on anti‐proliferative activity in A549 cells. A549 cells were treated with K‐563, cisplatin, etoposide, K‐563 plus cisplatin or K‐563 plus etoposide for 72 h, and the cell viability was determined. The results at concentration ratios of 1:15 (K‐563:cisplatin) (A) and 1:2 (K‐563:etoposide) (B) were represented as the percent of cell viability in drug‐treated cells relative to control cells. Each column represents the mean + SD of triplicate experiments

According to Singh et al^13^ who reported that the activated Keap1/Nrf2 pathway contributed to resistance to chemotherapeutic agents, cisplatin, or etoposide, in A549 cells, we examined the effect of K‐563 combined with cisplatin or etoposide on the viability of A549 cells. A single treatment of K‐563, cisplatin or etoposide inhibited the cell viability, but co‐treatment of K‐563 with cisplatin or etoposide inhibited the cell viability more strongly (Figure 4A,B). The CI values at 50% inhibition by K‐563 with cisplatin and K‐563 with etoposide were 0.74 and 0.52, respectively. These data with CI <1 highlight the synergism of K‐563 and either cisplatin or etoposide.

### 3.5 K‐563 inhibited the Keap1/Nrf2 pathway in an A549 lung cancer xenograft model

Finally, to investigate whether or not K‐563 inhibited the Keap1/Nrf2 pathway in tumors, we performed an in vivo study. We prepared human A549 lung cancer xenografts in SCID mice. Vehicle or K‐563 at 100 mg/kg was administered subcutaneously to the mice twice a day for 1 day. K‐563 did not reduce the body weight with 1 day's subcutaneous administration.

**Figure 5:**
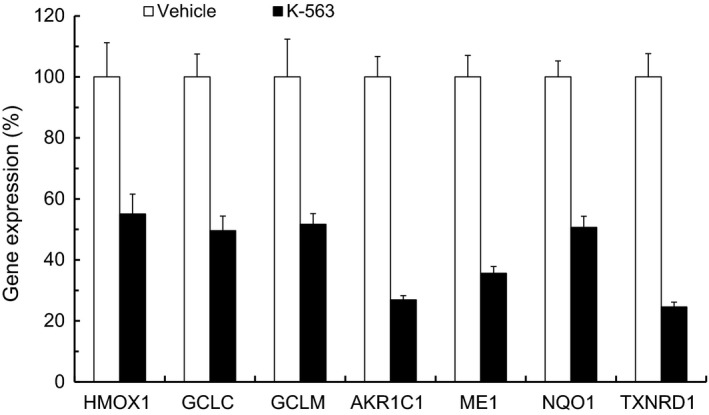
The expression of the Keap1/Nrf2 downstream genes in A549 tumors in mice treated with K‐563. K‐563 was administered subcutaneously to SCID mice xenotransplanted with A549 cells. After 24 h, tumors were collected from the mice and a real‐time RT‐PCR analysis was performed after mRNA collection. The mRNA expression was normalized to that of *GAPDH* mRNA. Each column represents the mean + SE (n = 5)

K‐563 inhibited the expressions of Keap1/Nrf2 pathway downstream target genes (*HMOX1*,* GCLC*,* GCLM*,* AKR1C1*,* ME1, NQO1,* and *TXNRD1*) in A549 tumors like in vitro (Figure 5). The mean expression of these seven Keap1/Nrf2 pathway downstream target genes in Figure 5 was 42%, which was equal to their mean expression at 3 μmol/L of K‐563 (48%) in Figure 2A. K‐563 inhibited the Keap1/Nrf2 pathway, including suppressing HO‐1 expression, GSH production, and ROS resistance, through the inhibition of Nrf2's transcriptional activity at 3 μmol/L (Figure 2B,C,F,G). These results suggested that similar inhibitory effects of K‐563 in A549 cells could be observed at 100 mg/kg in A549 tumors. Based on these findings, K‐563 was shown to inhibit the Keap1/Nrf2 pathway even in vivo by suppression of the transcriptional activity of Nrf2.

## 4 DISCUSSION

From our screening, the novel compound K‐563 was isolated from the culture broth of *Streptomyces* sp. 3728‐17. The structure elucidation of K‐563 revealed that this natural product was uniquely composed of alkylamine, phosphate imide of glycinamide, oxygenated cyclopentane, and nucleoside units. This composition has also been observed in 1100‐50^35^ and EM2487,^36^, ^37^, ^38^ making K‐563 the third compound in this natural product category. In the context of their activity, 1100‐50^35^ and EM2487^36^, ^37^, ^38^ have been reported as a nematocide and an anti‐human immunodeficiency virus (HIV) compound, respectively. In this study, we showed for the first time that K‐563 exerted Keap1/Nrf2 pathway inhibition and potent anti‐proliferative effects against cancer cells. The close similarity among these compounds suggests that 1100‐50 and EM2487 may also exert anti‐cancer effects through Keap1/Nrf2 pathway inhibition. However, K‐563 analogues of the different long alkyl side chain were only obtained from *Streptomyces* sp. 3728‐17. Other K‐563 analogues, including 100‐50 and EM2487, could not be obtained. Thus, the profile of Keap1/Nrf2 pathway inhibition by K‐563 analogues remains to be clarified. Further investigations will be required to clarify whether the other compounds in this natural product family do indeed induce Keap1/Nrf2 pathway inhibition or exert anti‐proliferative effects against cancer cells harboring Keap1 or Nrf2 mutations, as well as to discover new analogue compounds with Keap1/Nrf2 pathway inhibitory activity.

In the present study, K‐563 was identified as a Keap1/Nrf2 pathway inhibitor. However, we were unable to identify the molecular target of K‐563 on the Keap1/Nrf2 pathway due to difficulty synthesizing a chemical probe derived from K‐563. The activity of the Keap1/Nrf2 pathway is essentially exerted at the protein level of Nrf2, which is controlled by transcriptional expression and proteasomal degradation. The previously reported Keap1/Nrf2 pathway inhibitors, luteolin,^27^ brusatol,^28^ MLN385,^32^ and clobetasol propionate^33^ decreased the Nrf2 protein level by inhibiting *NFE2L2* expression or accelerating Nrf2 degradation. However, K‐563 exerted almost no effect on the Nrf2 protein level. This suggests that the mechanism by which K‐563 inhibits the Keap1/Nrf2 pathway differs from that of other Keap1/Nrf2 pathway inhibitors. The mechanism underlying K‐563 mediated Keap1/Nrf2 pathway inhibition had not been clarified yet. Therefore, clarifying the mechanism of K‐563 is of great interest and merits further experiments. Baba et al^37^ suggested that EM2487 inhibited the function of the transcription factor *tat* of HIV type I. Because the chemical structure of K‐563 is similar to that of EM2487, it was predicted that K‐563 might inhibit the transcription function of Nrf2. According to previous reports, EM2487 inhibited *tat*'s transcriptional activity but did not inhibit *tax*'s transactional activity of human T‐lymphotropic virus type I.^38^ We herein showed that K‐563 inhibited the reporter activity in A549/ARE‐Luc cells more strongly than that in A549/E‐cad promoter‐Luc cells. According to previous reports, E‐cadherin is regulated by several transcriptional factors, such as Snail, Slung, Twist, and ZEB1/2.^39^ Therefore, it was suggested that K‐563 did not inhibit the activities of these transcriptional factors. Further investigations of the target transcriptional factors of K‐563 may help elucidate the detailed mechanism by which K‐563 inhibits the Keap1/Nrf2 pathway.

K‐563 also exerted inhibitory effects on the Keap1/Nrf2 pathway in vivo. Based on the present findings, K‐563 may be a valuable lead compound as an anti‐cancer therapeutic. To investigate the anti‐tumor activity of K‐563, further in vivo studies to evaluate the anti‐tumor effects and combination effects with chemotherapeutic agents, observed in vitro, are needed. On the other hand, the in vivo toxicity of Keap1/Nrf2 pathway inhibitors, including K‐563, has not yet been evaluated. In Nrf2 knockout mice, Nrf2 deletion caused hemolytic anemia due to splenomegaly and spleen toxicity or resulted in impaired protection against xenobiotic toxicity.^40^, ^41^ Further in vivo studies in mice are needed to investigate not only the anti‐tumor effects, but also the toxicity of Keap1/Nrf2 pathway inhibitors, such as their blood toxicity and the detoxification of oxidative stress.

In conclusion, we discovered a novel Keap1/Nrf2 pathway inhibitor from *Streptomyces* sp. 3728‐17. The further investigation to find more potent derivatives from K‐563 or an investigation of the mechanism through which K‐563 exerts its activity is expected to lead to the development of anti‐cancer agents for Keap1‐ or Nrf2‐mutated cancer patients.

## CONFLICT OF INTEREST

The authors declare no potential conflicts of interest.
