# Supplementary figures and images for: The discovery and characterization of K‐563, a novel inhibitor of the Keap1/Nrf2 pathway produced by Streptomyces sp
Source: Cancer Med. 2019 Feb 8;8(3):1157–68. doi: 10.1002/cam4.1949 (PMC6434342; doi:10.1002/cam4.1949)

Supplementary Figure S1

A

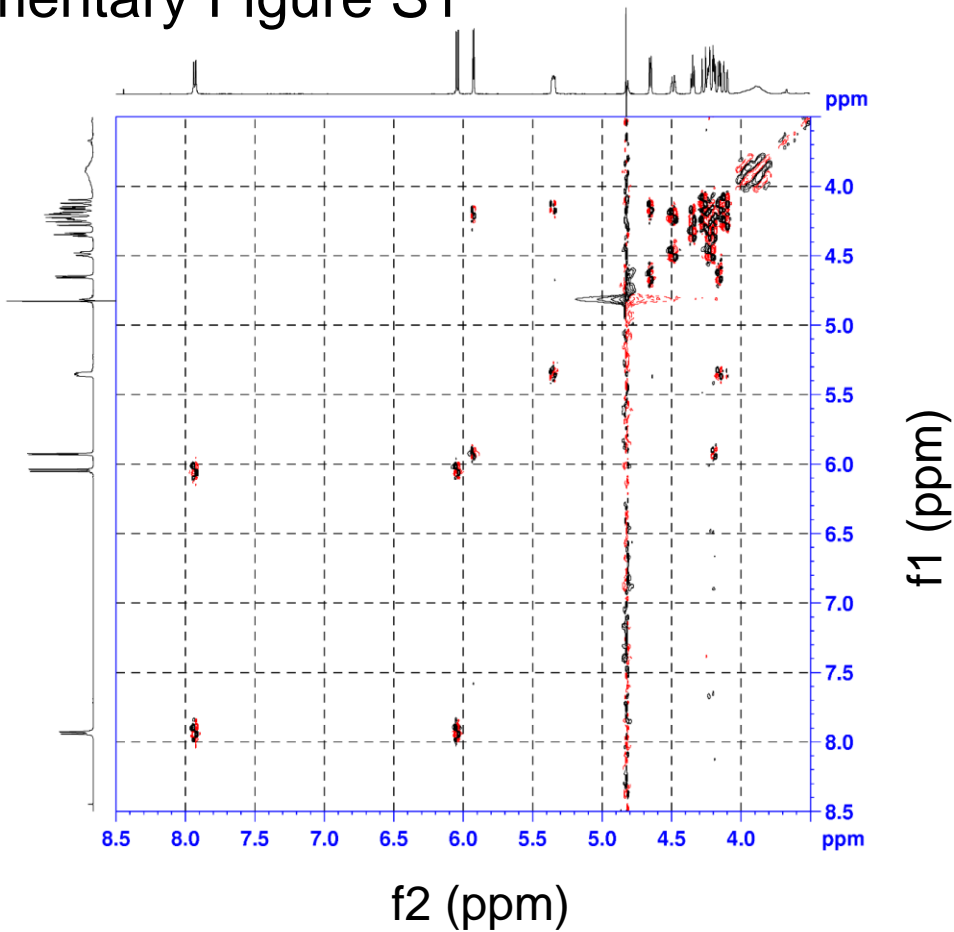

B

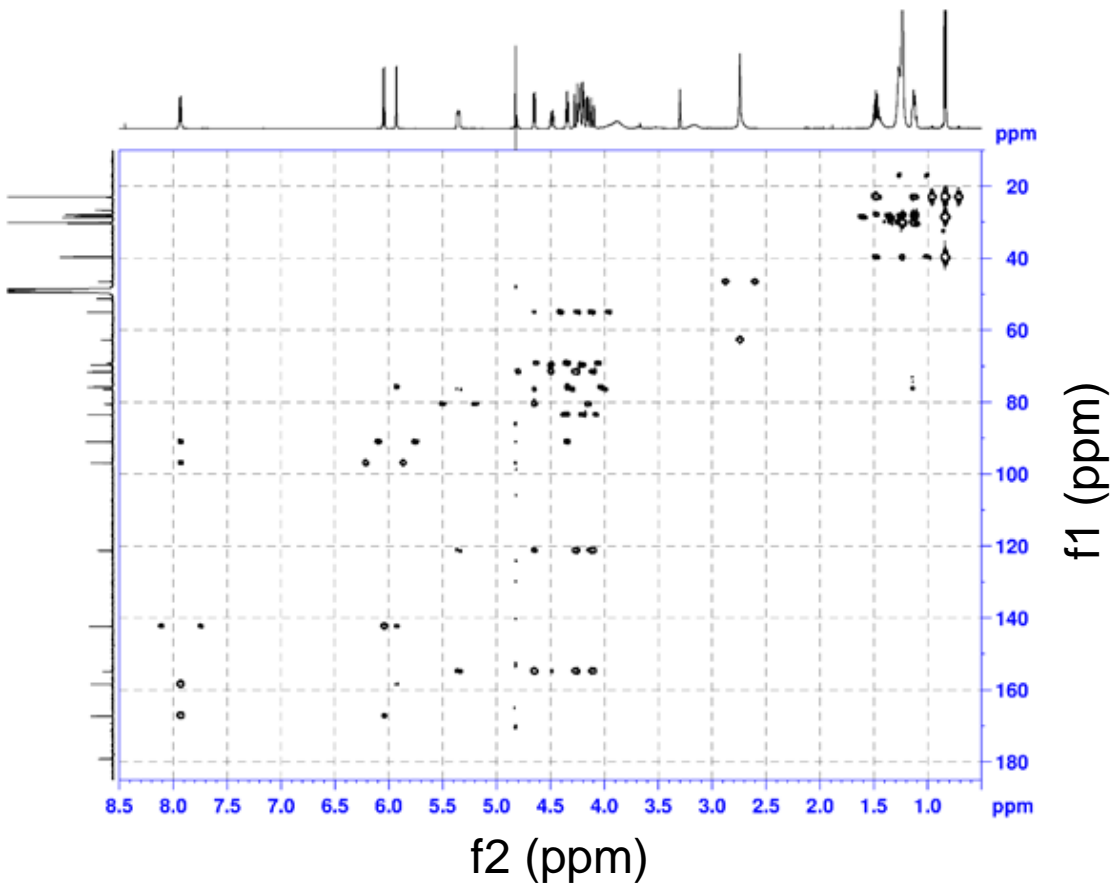

# Supplementary Figure S2

A

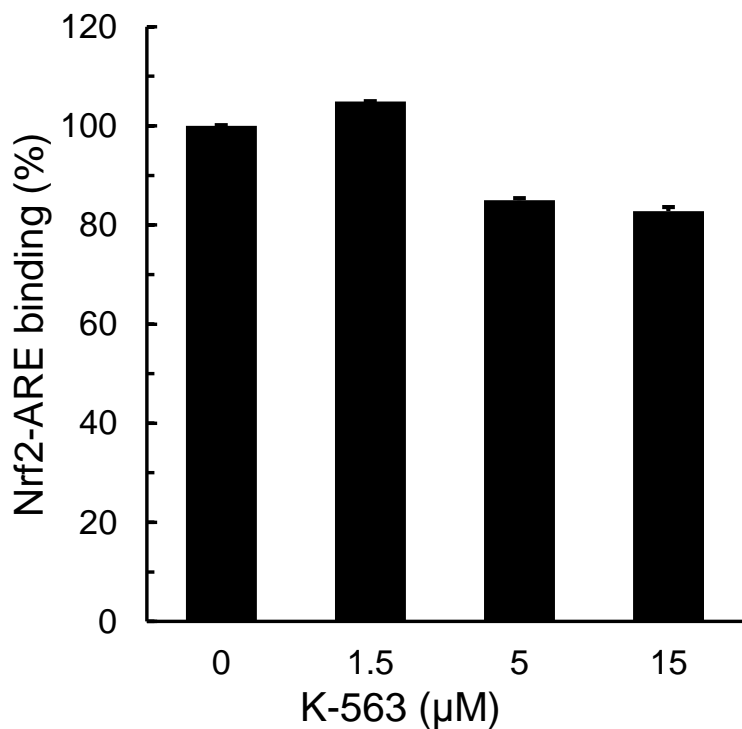

B

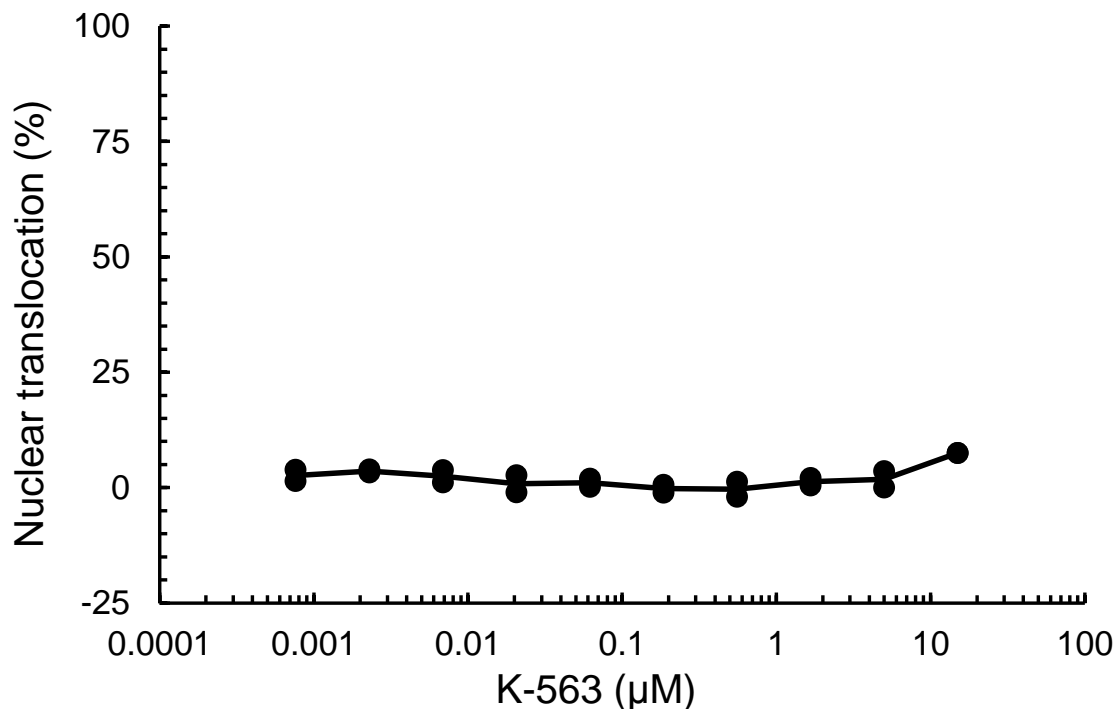

Supplement: Supplementary file 1 [file CAM4-8-1157-s001.pdf]
