# Supplementary material for: The discovery and characterization of K‐563, a novel inhibitor of the Keap1/Nrf2 pathway produced by Streptomyces sp
Source: Cancer Med. 2019 Feb 8;8(3):1157–68. doi: 10.1002/cam4.1949 (PMC6434342; doi:10.1002/cam4.1949)
Supplement: Supplementary file 2 [file CAM4-8-1157-s002.docx]

**Supplementary Methods:**

**Culture of *Streptomyces* sp. 3728-17**

1/2SR4 medium (0.5% glucose [Nacalai Tesque], 0.5% soluble starch [Nacalai Tesque], 0.15% Elrich bonito extract [Kyokuto Pharmaceutical], 0.25% yeast extract [Kyokuto Pharmaceutical], 0.25% Bacto tryptone [Wako], 0.1% KH_2_PO_4_ [Nacalai Tesque], 0.05% MgSO_4_·7H_2_O [Wako], 0.05% Mg_3_(PO_4_)_2_·8H_2_O [Nacalai Tesque]; pH 7.0) was used for seed culture for S*treptomyces* sp. 3728-17. After cultivation at 28 °C, fermentation was carried out in LBM4 medium (2% dextrin [Kanto Chemical], 1% γ-cyclodextrin [Junsei Chemical], 1% dry yeast [Oriental Yeast], 2% V8 vegetable juice [Campbell], 0.0005% CoCl_2_·6H_2_O [Sigma-Aldrich]; pH 7.0) at 28 °C.

**Extraction and purification of K-563 from *Streptomyces* sp. 3728-17**

After fermentation, the culture broth was centrifuged to separate the mycelia and supernatant. The mycelia were extracted with 50% aqueous 2-propanol (Wako) by gentle agitation with an auto-agitator (EYELA) for 1 h. The extract was centrifuged, and the mycelial pellets were extracted again in the same manner, after which all supernatants and mycelial extracts were mixed well to give a crude extract. We supplemented 4% of 500 mM ammonium acetate buffer (pH 9.0) to the crude extract to prevent hydrolytic degradation of K-563.

K-563 was purified by activity-guided column chromatography. A Diaion HP20 (Mitsubishi Chemical) was equilibrated with 10% methanol in 20 mM ammonium acetate buffer (pH 9.0). The 2-propanol concentration of the crude extract solution was adjusted to 10% and then applied to the Diaion HP20 column at a flow rate of approximately 1 L/h. The resin was washed with 5 L of 50% aqueous methanol supplemented with 4% of 500 mM ammonium acetate buffer (pH 9.0), and then K-563 was eluted with 5 L of methanol supplemented with 4% of the same buffer. The methanol concentration of the eluate was adjusted to 70% with water and then mixed well with *n*-heptane (Godo). The 70% methanol layer was collected and concentrated *in vacuo* until the methanol was removed. This solution was diluted with water so as to be frozen well at -40 °C and then lyophilized (VirTis, AdVantage) at -10 °C. The crude powder was dissolved at 30 mg/mL in 20 mM ammonium acetate buffer (pH 9.0) and subjected to further purification by preparative reversed-phase high-performance liquid chromatography (HPLC) under the following conditions: column, YMC-Actus Triart C_18_ (20 mm i.d. × 250 mm length; YMC); column temperature, ambient; detecting wavelength, 272 nm; mobile phase A, 20 mM sodium phosphate buffer (pH 9.0); mobile phase B, acetonitrile; elution program, 35% B (0 min) – 35% B (20 min) – 70% B (30 min); and flow rate, 12 mL/min. The HPLC fractions containing the purified K-563 were desalted using a small Diaion HP20SS column (Mitsubishi Chemical). Because the purified active compounds were stable in the following solution, the solvent was adequately selected depending on the purpose: DMSO (Wako), 20 mM ammonium acetate buffer (pH 9.0), 5 mM sodium phosphate buffer (pH 8.0), sodium phosphate-buffered saline (5 mM sodium phosphate buffer at pH 8.0 containing 0.9% NaCl), or 0.02 M NaOD (Sigma-Aldrich)/CD_3_OD (Merck Millipore) (1/1) mixed solution.

**HPLC and liquid chromatography with tandem mass spectrometry (LC-MS) analyses of K-563**

The concentration and purity of K-563 were confirmed by HPLC with the following conditions: column, YMC-Triart C_18_ (4.6 mm i.d. × 150 mm length; YMC); column temperature, 40 °C; mobile phase A, 20 mM ammonium acetate buffer (pH 9.0); mobile phase B, acetonitrile; elution program, 35% B (0 min) – 50% B (10 min) – 100% B (10.1 min) – 100% B (12 min); and flow rate, 1 mL/min. The molecular weight of K-563 was confirmed by LC-MS. The LC condition was the same as described above. The MS condition was as follows: inlet flow, adjusted under 0.5 mL/min by a post-column flow splitter; ionization mode, electron spray ionization (ESI); desolvation temperature, 450 °C; source temperature, 150 °C; desolvation gas flow, 600 L/h; cone gas flow, 50 L/h; capillary voltage, 3 kV; cone voltage, 10 and 30 V; and LC-MS system, HITACH pump L-7100, Waters System Fluidics Organizer, Waters 2998 PDA Detector, Waters 3100 Mass Detector.

**Nrf2 nuclear translocation assay**

PathHunter Keap1-NRF2 Functional Assay of K-563 was performed by DiscoveRx.

**Nrf2-ARE binding assay**

After K-563 treatment for 24 h, nuclear Nrf2 proteins in A549 cells were collected by Nuclear Extract Kit (Active Motif), according to the manufacturer’s instruction. Nrf2-ARE binding assay was measured by TransAM Nrf2 (Active Motif). Absorbance was measured as readout, by using SpectraMax 340PC (Molecular Devices). Each Nrf2-ARE binding level was normalized by the values obtained from DMSO-treated sample.
