# Supplementary material for: The discovery and characterization of K‐563, a novel inhibitor of the Keap1/Nrf2 pathway produced by Streptomyces sp
Source: Cancer Med. 2019 Feb 8;8(3):1157–68. doi: 10.1002/cam4.1949 (PMC6434342; doi:10.1002/cam4.1949)
Supplement: Supplementary file 3 [file CAM4-8-1157-s003.docx]

**Supplementary Figure Legends:**

**Supplementary Figure S1. DQF-COSY (A) and HMBC spectrum (B) of K-563 in 0.02 M NaOD/CD3OD (1/1).**

**Supplementary Figure S2. Effects of K-563 on Nrf2-ARE binding and Nrf2 nuclear translocation.** A, A549 cells were treated with K-563 for 24 h and Nrf2-ARE binding assay (Active Motif) was performed. Each column represents the mean + S.D. of triplicate experiments. B, Nrf2 nuclear translocation was measured with PathHunter Keap1-NRF2 functional assay (DiscoveRx) after 18 h of K-563-treatment. Each point represents the individual data of duplicate experiments.
